# Parental Perception of Children’s Online Behaviour: A Study on Ethnic Communities in Australia

**DOI:** 10.3390/ijerph20075342

**Published:** 2023-03-30

**Authors:** Ahmed Imran, Nilufa Khanom, Azizur Rahman

**Affiliations:** 1 School of Information Technology and Systems, Faculty of Science and Technology, University of Canberra, Bruce, ACT 2617, Australia; 2 Victoria University Business School, Victoria University, Melbourne, VIC 3000, Australia; 3 School of Computing, Mathematics and Engineering, Charles Stuart University, Wagga Wagga, NSW 2678, Australia

**Keywords:** parental perception, children cyber safety, cyber risk, ethnic communities, children online, multi-cultural

## Abstract

The overwhelming growth of the Internet in all spheres of life poses new challenges for young children growing up in the digital age, with potential short- and long-term ramifications. Parents have an essential role in the development of the attitudes and behaviour of their children. However, studies indicate that adults are not adequately mitigating the range of cyber risks that children face and that parent-oriented solutions are simply inadequate. This study attempts to fill research gaps in the status and nature of parents’ perceptions of the online use of their children in Australia based on their ethnic background. This study adopted a mixed-method approach, surveying 204 parents from different ethnic communities in Australia followed by 16 in-depth interviews and three focus-group discussions. The results indicate that parents’ perceptions of online risk for children differ based on their ethnicity, cultural adaptation, gender, and age. Parents from multicultural societies are less equipped to deal with cyber threats that their children face and are ill-equipped to monitor and mitigate the risks posed. The results of this study have important policy implications, from deepening our understanding of the nature of the problems to facilitating the development of short- and long-term strategies, appropriate information systems, policy guidelines, and interventions.

## 1. Introduction

The growing use of the Internet by children and their online behaviour are becoming a deep concern, particularly amongst parents of the digital era. Over the last decade, the discourse on technological development has been primarily framed in favourable terms, such as progress, innovation, benefit, and opportunity [1]. The educational benefits of technology have been taken as evident, with many developed nations enacting policies to provide children with the technologies deemed necessary for their future success in the global marketplace. Parents are keen to ensure that they acquire the required technical skills for the jobs of the future [2].

In Australia, the digital opportunities have been framed in terms of the educational potential of digital media, where 97% of households with children under 15 years of age have access to the Internet, with an average of seven devices per household. Many of the dangers have typically been presented as a discrete set of risks, including cyberbullying, exposure to inappropriate content, and a range of adverse health impacts [3].

Australia stands as one of the most multicultural nations on the planet. According to 2021 ABS data, over 7.6 million migrants live in Australia; 29.8% of the population were born overseas, coming from nearly 200 countries, with more than 300 languages spoken in homes, over 100 religions, and more than 300 different ethnic ancestries [4]. As such, Australia has several large and small ethnic groups. These groups have a ‘sub-culture’, where ethnicity enhances the sense of belonging and togetherness of a particular community or sub-cultural group [5]. In an increasingly diverse society, these demographic differences may become more defined and widespread in future years The values, perceptions, and child-rearing styles of parents vary according to their diverse beliefs, values, backgrounds, and cultures [6,7,8,9]; thus, parents’ perceptions of the online behaviour of their children may vary within Australia. The attitudinal differences between children and parents and their social environment have become a severe concern for parents in ethnic communities in Australia [10].

Parents’ roles in mediating the online activities of their children and minimising risks while maximising opportunities have been widely addressed by researchers in the field [11,12,13,14,15,16,17,18]. However, parents’ experiences and perspectives on this issue remain relatively underexplored [14], particularly the ethical views of parents in Australia. Furthermore, few studies have focused on ethnic communities in Australia and parental perception of internet use, e.g., the use of digital and mobile technologies in Culturally and Linguistically Diverse (CALD) young people [12]. 

It is thus essential to look at parents’ perception of their childrens’ internet use within ethnic communities in Australia, particularly with Australia being a multi-cultural country. This research attempts to uncover the issue by examining the effective interaction of parents’ backgrounds with their perception of the Internet behaviour of their children to see how this varies amongst different ethnic communities in Australia.

## 2. Literature Review

Parents and carers bear primary responsibility for ensuring the safety and well-being of their children [17]. Considerable research has been conducted on cyberbullying, cyber safety, and cyber-smart parents, as demonstrated through the development of online cyber safety tools in Australia [11,19,20]. However, there is a considerable gap in the research on parental perception and awareness, especially through the lens of parents from different ethnic and multicultural backgrounds [12,21]. This research attempts to address these issues by discussing the literature on childrens’ internet use, the risks of internet use, parental perceptions and awareness of childrens’ internet use, and, finally, the cultural perspective of ethnic parents and their concerns about internet use.

### 2.1. Children’s Internet Access

Children have wide access to the Internet in almost all developed countries, including Australia, the USA, and Europe. For example, in Australia, for households with children aged under 15 years, 97% have access to the Internet [22]. Young people aged 15–17 years old have the highest number of users at 99%. They spend an average of 18 h per week online [23]. Access to the Internet is similarly high in the USA. In 2018, some 94% of 3–18-year-olds had home internet access: 88% had access through a computer and 6% had access only through a smartphone [24].

### 2.2. Risk Perception

Risk perception is the “cognitive process that rests on the information that each person has on certain issues … and that each one processes by organizing their value judgments”, which will condition their behaviour [25]. The perception of risk is relevant to other indicators, such as the child-rearing techniques used by parents, the time children spend on Internet, the behavioural patterns of children, their dependency on the device, and the type of digital behaviour involved [26].

A study by Ramos-Soler, López-Sánchez and Torrecillas-Lacave [26] confirmed that age, parental guidance, and behavioural variables (e.g., level of confidence) are significantly related to the perception of risks. For example, the prudent behaviour of school students on social networks is consistent with their perception of risk when their parents guided their behaviour. Moreover, friendly and self-confident school students acknowledged that they used to discuss their internet use with their parents. At the same time, the connected and independent group (aged 17 years old) were also aware of the risks but never asked about or discussed such issues with their parents. The group of confident players are identified as having a lack of risk perception as they do not identify behaviour such as harassment, being the victim of blackmail, or receiving offensive messages, among others, as risks of using the Internet [26].

### 2.3. Risk in Internet Use

With the massive proliferation of the use of the Internet in every aspect of our lives, exposure to risks is also increasing in varied forms [27,28], including the risk of cyber-aggression or cyber victimization [29]. These risks range from gaming addictions, danger from meeting strangers, social isolation, and cyberbullying all the way to severe consequences such as suicide, cyber terrorism, and so on. 

In Australia, children may face several negative experiences when accessing the Internet. Recent research by the eSafety Commissioner found that just over 4 in 10 teenagers (between the age 14 to 17) had at least one negative online experience in the 6 months prior to September 2020, with this increasing to over 50% in those aged 14 to 17. The top five negative online experiences of teens included: being contacted by a stranger or someone not known to them—30% (26% of males and 35% of females); receiving inappropriate, unwanted content such as pornography or violent material—20%; being deliberately excluded from events/social groups—16%; receiving online threats or abuse—15% (18% of males compared with 11% of females); and having things said online to damage their reputation—15%. Moreover, almost one-third (30%) said their negative online experience related to bullying at school [11]. Furthermore, the ABS study [23] found that in 2016–2017, for children aged 5–14 years, 14% of connected households stated a child had been exposed to inappropriate material and 5% of those households said a child had been subject to cyberbullying [22]. One of the significant risks surrounding the Internet is ‘cyberbullying’, which affects the family environment and disrupts family relationships, being less authentic than relationships cultivated offline [15]. ‘Cyberbullying’ and ‘bullying’ were identified as a big problem by 53% of Australian parents [30]. Other potential threats posed by the Internet are pornography, violence, unsupervised social relations, and privacy and security issues [31].

The risks of using the Internet are common around the world. For example, UNICEF (2022) [32] reported in South Africa that more than 50% of children and young people were exposed to sexual content online, 22% in Italy and Uruguay were exposed to content on self-harm, and 35% of children surveyed in Italy and Uruguay said they were exposed to hate speech [32].

### 2.4. Parental Concerns about Risks

Australian parents are concerned about use of the Internet with respect to several issues, such as digital media disrupting learning and development as children are compromising their inherent intellectual capacity and critical thinking skills are being altered by using the Internet, including a lack of self-discipline resulting in ‘intellectual laziness’ and ‘stunted’ mental abilities [14]. The three most common concerns cited in the eSafety research [11] for Australian parents were: exposure to inappropriate content other than pornography (38%), contact with strangers (37%), and being bullied online (34%) [21]. Cyberbullying is the second biggest health concern for Australian parents, after screen time [30]. Furthermore, parents are highly concerned about the social reputation of children being affected through the sharing of inappropriate content (e.g., sexual images) on social media and the erosion of the social skills of young people, with the latter disrupting family relationships [15]. Earlier studies found that parents are concerned about the increased mediation of friendships and other forms of social isolation and familial relations [33].

### 2.5. Parental Awareness and Understanding of the Risks of Internet Use

Parental awareness about internet risk is lacking in different countries and cultures. For example, some European studies revealed that parents are largely unaware of their children’s engagement in risky online activities, including accessing violent or pornographic web content and experiences with cyberbullying [34,35]. Most parents in the Netherlands were unaware of whether their child was engaged in cyberbullying as a perpetrator or as a victim [35], and parents do not always know if their children have been bullied [36].

According to an ACMA study [10] in Australia, most parents appear to have a good understanding of how their 8–11-year-old children use the Internet for social networking, playing games, and doing homework [10]. Furthermore, parents are more likely to under-report the online activities of their older child [10]. These trends indicate that while parents are concerned about their children’s online behaviour, certain variables, such as age, distort their perception of their children’s online use.

Parental awareness differs from country to country and depends on the level of knowledge of parents about internet use. For example, a recent Malaysian study found that parental awareness of cyber threats still needs to be improved to promote cyber safety [37]. In many cases, Malaysian parents were unaware of their children’s unregulated access and exposure to inappropriate online sites, subjecting them to cyber threats. Regarding victimization, the study found that 11.8% of the parents believed that their child was bullied online, against 22.9% of the children reporting this. Another study conducted in the US by Byrne et al. (2014) [34] found that around one in three parents accurately knew whether their child’s involvement concerned victimization from cyberbullying or its perpetration.

A study by Symons et al. (2017) [38] found that parental awareness and self-perceived knowledge of their children’s online activities differ for mothers and fathers. For example, mothers were more reasonable than fathers in saying that they knew with whom their children had shared personal information, but they were not more likely to say that they knew which websites their children visited. On the other hand, fathers are more likely to indicate that their daughters have been the victims of cyberbullying. The study by Liau et al. [39] found that parental knowledge about adolescents’ frequency of internet use was lowest among fathers regarding the internet use of their sons. Mothers and fathers were also more likely to indicate that their sons had viewed pornography than their daughters [38]. Their study also found that mothers did not have more accurate knowledge than fathers. Both parents were less likely to know accurately about the occurrence of content risks (watching pornographic content and watching violent content) for girls [38]. Furthermore, what is known about parental knowledge is mainly derived from reports from the mother, whose knowledge tends to be superior to that of the father [38]. Overall, it can be concluded that parental knowledge about children’s online behaviour tends to be suboptimal.

### 2.6. Ethnic Culture and Cultural Influence on Parental Perspectives

Ethnicity depicts the commonality of shared norms, beliefs, and values transmitted to each generation that are deeply rooted in culture [40]. Culture is intertwined with race and ethnicity—and the identities these lead to—as well as being distinct from them, which adds to the difficulty of unravelling what culture is [41]. Researchers insist that ‘defining culture is one of the most difficult’ and controversial tasks as culture is a construct that defies easy explanation [42,43]. Moreover, when culture is associated with a specific group or ethnicity, this has conventionally been race-related. Race or ethnicity is a label typically imposed by bigoted societies—something by which people are categorised and often disregarded [41].

Several cross-cultural parenting research reports have drawn an association between childrearing aspects (such as parenting goals) and the features of ‘individualistic’ and ‘collectivist’ cultures [44]. Individualistic (‘Westernised’) cultures are considered to value personal freedom, independence, and the capacity to get things done on one’s own. As a result, they encourage children to act autonomously and demonstrate initiative from an early age. On the other hand, collectivist (Eastern and sub-Saharan) societies place a sense of community and broader responsibility beyond individual interests. In collectivist cultures, children are encouraged to focus on group issues, and parents prepare them to take on more responsibilities for their family and the community. Through these processes, the independent or interdependent value systems of individualism and collectivism, respectively, shape family interactions and expectations [45], parenting theories and styles [46], and educational aspirations [47]. In addition, researchers generally agree that parents hold childrearing goals that are consistent with the expectations held by the culture with which they affiliate [44].

Cultural variations in parenting beliefs and behaviour are also evident in the study of the Australian Institute of Family Studies (AIFS) (2015) [48] in Australia. The research found differences in parenting goals that reflected parents’ individualistic and collectivist cultures [48]. For example, independence and social skills were valued more among Anglo parents who belong to ‘individualistic’ cultures than Vietnamese and Somali parents (who are from ‘collectivist’ societies); meanwhile, conformity was valued more by Vietnamese parents than that of Anglo parents [48].

Parents’ cultural dimensions influence their interactions with and attitudes towards their children. For example, Anglo-American and Canadian parents from individualistic cultures [49] tend to encourage and support appropriate behaviour when children are toddlers, as they believe children can control their behaviour from a young age. In contrast, parents from collectivist cultures [49], such as African American and Hispanic cultures, tend to have more parental control over the child’s environment and how they behave until middle childhood [50]. Such parental behaviour could be equally applicable to the children’s other activities, including internet use and online behaviour.

### 2.7. Parental Cultural Influence on Children’s Online Behaviour

Parental attitudes and cultural backgrounds have an effect on shaping children’s online behaviour. Research shows that internet users’ behaviour and attitudes toward the Internet vary across individualistic and collectivistic cultures [8]. Parental mediation of children’s internet use also varies significantly [9]: for example, the study of Chan and Shen (2004) [51] suggests that Chinese parents tend to focus on children’s education and health, which is derived from their ‘collectivist’ background culture and attitudes. Thus, they prefer protection through restrictions on their children’s internet use rather than mediation [51]. Similarly, Hong Kong Chinese parents are generally more concerned about the harm incurred through internet use than their counterparts in Western countries, which also reflects their cultural background and attitudes [51]. Chan and She (2004) [51] also reported that Chinese parents have mixed and contradictory feelings toward the Internet. While the intention to let children “find their way in the world” directly conflicts with the parental purpose of protecting them from harm, since Chinese parents strongly hold ‘collectivist’ cultural views [52], they also encourage their children’s internet use to better their education, as Chinese parents focus more on children’s education and health [51].

There is scant research on cognitive psychology and parental attitudes, although several studies have revealed different parental attitudes to children’s internet use. For example, in the USA, White and Asian/Pacific Islander children are more likely to use the Internet at home (64% and 63%, respectively) than Black or Hispanic children (49% and 44%, respectively). Jewish parents favour the co-use of Facebook with children and are more familiar with monitoring technologies than Arab parents [53]. Most parents in Singapore do not exercise adequate supervision of their children’s activity on the web [39]. It is assumed that these differences have resulted from differences in cultural cognitive knowledge.

The parental concerns of CALD parents in Australia are also different. The 2018 Youth and Digital Dangers report highlighted that Australian children from a CALD background were less likely to report a negative online experience to their parents [11]. When it came to CALD parents, they were shown to be significantly more aware of their child’s negative online experiences than those from a non-CALD background (39% vs. 24% for non-CALD parents) [21].

## 3. Method

In this study, a mixed methods approach was followed to explore the influence of culture and ethnicity on parental perception, first through an online survey followed by interviews and focus groups consisting of parents from different communities. Such an approach is advocated to address complex phenomena, particularly to unveil interrelations among contentious issues [54]. According to Greene (2008, p. 20) [55], ‘a mixed methods approach to social inquiry distinctively offers deep and potentially inspirational and catalytic opportunities to meaningfully engage with the differences that matter in today’s troubled world, seeking not so much convergence and consensus as opportunities for respectful listening and understanding’.

Accordingly, the first phase involved an exploratory (descriptive) survey to gauge parents’ understanding and perception of their children’s online use. An advantage of using such a survey is that it can accurately document the norm [56]. In the second phase, a qualitative study consisting of sixteen individual interviews and three demographic focus group discussions (FGDs) was carried out. The objective of the second phase was to complement the survey findings and to expand our understanding of the perceptions of the parents, particularly from multicultural ethnicities. Participation in this research was open for eligible parents of children aged between 4 and 18 years living in Australia, which represents a wide section of South Asian and other ethnic and cultural backgrounds. Ethical clearance was obtained prior to the field study from the Human Research Ethics Advisory (HREA) Panel of UNSW Canberra (HREA Panel Ref: A-15–26 and date of approval 27 May 2015). All participants signed a consent form and were provided with information about the nature of the research and their confidentiality rights, either verbally or through an information sheet.

### 3.1. Phase 1: Survey

#### 3.1.1. Sample and Settings

Based on the study design, targeted population consists of parents from different ethnic communities living in Australia with children aged < 18 years. We distributed the survey questionnaire to 600 participants following the simple random sampling technique. A total of 205 completed survey forms were collected between August 2015 and January 2018, with a response rate of 34.16% (=205/600) using Survey Monkey (Gold plan). Seven individuals did not provide complete information in the survey; therefore, a sample of 200 respondents’ data was considered valid for analysis for the study. (See sample profile in Section 4.1 and also in Table 2.)

#### 3.1.2. Measurement

Survey participants were asked questions relating to parents’ awareness of the Internet, parents’ perception of risks associated with the use of the Internet, parents’ trust and beliefs about their children’s internet use, and their personal data. Most of the survey questions out of total 39 questions in four sections were multiple-choice, with some requiring an answer on a Likert-type five-point scale. The survey questions were developed, improved, and verified based on previous findings and also through consultation with research panel experts. The questionnaire was prepared in English as the target audience of the survey was Australians who understand at least basic English. Survey procedures were designed to assist in maximizing the response rates, and the questionnaire was disseminated as both an online and a paper version. A summary of the survey questions is provided in Table 1.

### 3.2. Statistical Analysis

In analysing the survey data, both descriptive and multivariate statistical analyses were carried out to measure the links between the composite risk perception of cyber risks faced by children and some selected significant characteristics of the participants. Bivariate analyses using cross-tabulations were performed to assess the participant’s composite risk perception and various categories of the selected variables were considered for the multivariate analysis. The significant determinants were explored using Pearson’s Chi-squared test. Multinomial logistic regression modelling [57] for multivariable analysis was carried out to determine the influence of some important covariates on the likelihood of experiencing the composite risk perception, such as the variables ‘some risk’ and ‘real risk’ considered in this research.

The significant relationships between the variables and their effects were quantified by calculating the odds ratios with the 95% confidence interval measures. The odds ratio (OR) in favour of some and real form of risks was computed for the selected group of covariates to suggest how many times the group of interest more probably belongs to the target group compared to the reference group, i.e., ‘no’ risk. Moreover, the −2 Log Likelihood-based Chi-squared test was employed to check the statistical significance of the fitted model. Further details about these methods and analysis techniques are available in the existing literature [58,59]. The IBM SPSS version 26 (IBM Corp, New York, NY, USA) and R (i386.3.6.2, R Foundation, Indianapolis, IN, USA) software were utilised in all statistical analyses.

### 3.3. Phase 2: Focus Group and Interviews

Focus group participants were mostly selected from the survey respondents who expressed their interest for an in-depth follow-up interview or FGD. Participants largely came from South Asian ethnicity, such as from Myanmar (Burma), Afghan, Indian, Sri-Lanka, Pakistan, Bangladesh, India and the Philippines, who share similar values and characteristics based on Hofstede’s [49] classification. Of the interview participants, at least 50% have lived in Australia for more than 3 years, 50% had several children, and the remaining 50% had one child. Interview participants were asked semi-structured questions relating to their perception of internet risk, parental awareness of their child’s internet use, parental knowledge of actions that lower risks associated with internet use, and parental ability to implement actions to eliminate risks associated with internet use. See Appendix A. The focus groups responded to two statements—the first about the perception of and views about children’s online behaviour and associated risks and the second about strategies to prevent risks. The focus group and interview data were analysed both through manual coding techniques and computer-aided analysis using NVIVO (11, QSR International, Massachusetts, MA, USA) software. The coding and categories were developed keeping synergy with the survey data.

## 4. Findings

The major findings from both phases are appended below:

### 4.1. Survey Respondent Demographics

Each survey participant had a median of two children under the age of 18, and the majority (75%) of parents did not indicate that they had any other children who were over the age of 18. From the sample, 34% were male, 47% were female, and 19% did not mention gender. The most common age range of the parents surveyed was 45–55 years old. Survey participants had a range of ethnicities but were predominantly from South Asian countries (48%), comprising India, Pakistan, Bangladesh, Vietnam, China, Nepal, Myanmar, and Afghanistan. The remaining comprised Caucasian, Anglo-Saxon, and Middle Eastern ethnicities. The majority of respondents said that English was their medium of instruction in their educational institution, with close to a 50% split between those respondents born overseas and those born in Australia; this means the survey sample is not reflective of Australia’s 29.8% of citizens born overseas [4], but rather provides a snapshot of the overseas-born population.

### 4.2. Risk Perception

The background characteristics of the participants and their percentage distribution by the composite risk perception index are presented in Table 2. Of the 200 parents surveyed to gauge their perception of their children’s cyber safety, nearly one-half (48.5%) were of South-East Asian ethnicity. The gender distribution of the participants was fairly even, with 52% of the respondents being male. Almost two-thirds of the respondents had an undergraduate to a doctoral level degree, and 60% of the participants accessed the Internet using an iPad. In addition, most of the respondents (81.5%) understood cyberbullying and 57.5% were familiar with their school’s cyber policy and legislation. Three out of each four respondents (75.5%) were okay with their children using the Internet for contacting friends and family, while 69.5% were not happy if a child accessed the Internet for gaming. Moreover, most of the participants (94%) believed that internet access exposes their children to danger/risk.

The bivariate analysis results reveal that a significant proportion of parents who think their children face no real risk online or just some risk happen to be male (76.9% and 69.2% respectively), whereas a larger percentage of those who have a risk perception in the real risk category are female (57%) (see Table 2). A significantly high percentage (54.8%) of parents with South-East Asian ethnicity reported a real risk perception, which also increased with parents’ educational levels. Specifically, 57.7% and 70.4% of the parents having undergraduate to doctoral level degrees reported some risk and real risk, respectively, for their children when accessing the Internet.

Regarding knowledge about cyber risk variables, a majority of the parents who understood the danger of cyberbullying also felt that their children faced real risk online (86.7%). However, almost half (46.2%) of those who perceived no risk also claimed to understand cyberbullying. Parents who were familiar with their children’s school’s cyber policy and Australian legislation surrounding online safety for children make up the many parents who believe that their children are not at any real risk (92.3%), indicating that they may have high confidence in existing policies and legislation.

Moreover, it is interesting to note that amongst parents who had the highest risk perception, 80.7% believed contacting family and friends is an appropriate reason for children to use the Internet, while only 27.4% believed playing internet games is an appropriate reason for children to access the online environment. Moreover, of the parents with a solid belief that there is a significant danger or risk associated with access to the Internet, about 92.3% and 97.8% of those respondents reported some risk and real risk, respectively. It is worth mentioning that a small percentage of parents (6%) who do not consider their children to be in danger when using the Internet do acknowledge some dangers, such as cyberbullying, contacting strangers, and disengaging with the real world, while none of them seem to perceive the loss of privacy or security as dangers.

Using multivariate modelling, we considered multinomial logistic regression analyses to investigate the significant impacts of selected factors on the moderate and severe risk perception around children’s cyber safety. The results are presented in Table 3. Findings reveal that the male respondents were 85% (i.e., OR: 0.15; 95%CI: 0.02–0.87; *p*-value < 0.05) less likely to report feeling a significantly real risk for children in accessing online environments than their female counterparts, illustrating a very different level of risk perception between these groups. Compared to other ethnic groups (e.g., Anglo-Saxon, Caucasian), the South-East Asian respondents were 5.97 times more likely to feel that there is a real risk for children in cyberspace. Moreover, parents who did not complete high school education were about six times more likely to feel some risk in the cyber experiences of their children than those with an undergraduate to PhD level degree. Likewise, respondents who have access to the Internet with an iPad were almost seven times more likely than their counterparts to report feeling some risk for their children, with a *p*-value < 0.05.

Concerning knowledge of cyber risks variables, when compared to those parents who understand cyberbullying, parents without knowledge of cyberbullying were 89% and 92% less likely to report some risk and real risk, respectively. However, parents who are not familiar with their school’s cyber policy and Australian legislation were 11.7 times more likely to consider that there is some risk and 6.7 times more likely to consider that there is a real risk for children with internet access. Similarly, parents who do not feel that their children can use the Internet for contacting friends or families were 77% less likely to say that there is a real risk for their children than their counterparts. Respondents who do not consent to their children using the Internet for playing games were 4.1 times and 4.6 times more likely to state there is some risk and real risk to the child than those parents who consent to their children playing games on the Internet. Moreover, parents who do not believe that there are dangers on the Internet were 83% less likely and 94% less likely to feel that their child is at some risk and real risk while accessing the Internet compared to parents who think there are dangers on the Internet.

### 4.3. Areas of Concern for Parents

Parents’ understanding of their children’s use of computers was mostly limited to school activities, entertainment, computer skills, information gathering, and games. Only 39% of parents thought that their children used the Internet for socialising, which may be far different from their actual use. While parents were split in defining excessive use of the Internet for themselves, the majority considered even 1–6 h as excessive use for their children. This clearly showed their concern about children spending time on computers.

Major areas of concern revealed from the qualitative study are discussed below, which will further augment and complement the survey findings.

#### 4.3.1. Disengagement from Collective Social Life and Values

The concern about disengagement from collective social life was strongly reflected by the majority of the interview and focus group participants. Most parents from ethnic Asian and other cultures come from a collective society where close family ties and social bonds are an integral part of their life and upbringing. In those societies, new online access and communication appear to be unwanted intruders disrupting their expectations of their children. Disengagement and lack of interest in real-life activities, including outdoor games, family tours, and programs are turning them “anti-social”, as defined by some parents. Some parents in particular were found to be very concerned about their family life and values, as expressed by the parents:

My boy is losing interest in real-life activities (Interviewee-2); “I am very worried; the Internet is good, but it is also very bad sometimes” (Interviewee-4); “the Internet is making children very emotional and unrealistic about the practical world” (Interviewee-9); “Children are becoming less interested in family times and other family social gatherings, that will affect the family values and bonds”

(FGD-2)

There was a deep concern and fear of losing traditional values and practices among extended kinships among some ethnic parents. There was a fear of learning inappropriate culture, behavioural norms, and language deemed not acceptable to their own culture. For example, one interviewee said that his kids do not like to talk to their relatives:

They don’t like to come out of their room—their world. They don’t like to do any socialisation with other family members, or our friends when they come to visit us. They don’t even sometimes say ‘hi–hello’ to our family friends and relatives. Sometimes, it sounds like they don’t know what to say or how to talk to their friends or relatives

(Interviewee-5)

The issue I see when you send your kids to the playground to play, they physically see the other kid. You get to see them as well so where you can judge who she should mix with or not. You can see the risk but when you are online, you don’t see that. From my point of view, seeing is believing but I can’t apply that online. So, there is a significant amount of risks so what I would do probably when my daughter gets to that age I would limit her access through this sort of application where she gets the opportunity to mingle with other people whom she doesn’t see

(Interviewee-2)

These fears of the unknown and uncertainties without clear understanding and knowledge often lead to unwanted stress, anxiety, and broken relationships between parents and children.

#### 4.3.2. Watching Appropriate Content and Learned Behaviour

Some of the participants had strong religious and conservative views. Free access to the Internet exposes many areas of concern, such as photos, movies, and porn that are not culturally appropriate for certain families and societies. Some also attribute this to sources of violence, sex, and other offences, including terrorism. As one interviewee expressed regarding his concern about the numerous risks associated with using the Internet:

I see risk as social risk, cultural risk, and financial risk. Religious risk is if they are playing with their friends online who belong to some kind of notorious parties … financial risk is the information about my bank statements and my debit cards and my credit cards because I am doing my business … social or cultural risk if they are surfing more and more on the Internet it creates a social distance between us and our children

(Interviewee-3)

These parents believe that their children are learning inappropriate language and culture that are not acceptable in their society and religious beliefs. For example, the Afghan community said:

Through the Internet, our children are learning inappropriate cultures and languages that are not accepted by our own cultures, they are watching inappropriate content, e.g., photos and movies, that are not accepted by our own culture and particularly in our religion

(FGD-3)

Another South-Asian community member noted that:

Children are copying unethical behaviour from online friends and online dangerous groups; they are following inappropriate culture, and are exposed to violent games and activities, watching inappropriate content, e.g., photos and movies on terrorism, and radicalisation. That are not leaning good things always

(FGD-2)

Learned behaviour from online characters/avatars is likely to influence them and may have long-term impacts on their character-building. Children can be allured by strangers and dangerous persons or groups, including radicalisation. For example, one parent mentioned:

Whatever they (kids) see they put it in their minds. So, while they are using the Internet and we are not keeping an eye … they might be watching something very violent which may develop some violence in their mind. That’s one of the things and the other thing is … you know related to the adult themes.

(Interviewee-11)

The cartoon they watch mostly these very much deviate from real life; these are not in real life. They seem, they are always behaving differently, like dreaming, as they watch cartoons.

(FGD-5)

So, ads are not a risk for me, but the ad has a bad effect—ads could have a bad impact on her behaviour she has like you know she might fall in love with some sort of toy they put through in the advertisement through the cartoon. That could be a risk but I don’t see that as a major risk … When she watches cartoons, they try to sell a lot of toys through those cartoon advertisements and she attempts to pick them up and talked to me about them and see whether she can get hold of them from the shop.

(Interviewee-3)

Sharing private matters, particularly family issues, with strangers and online friends, is seen as a serious breach for some parents. For example, one parent said:

[Through online] that are making friendships with strangers and unwanted persons. And sharing private matters, particularly family privacy with unknown persons that is not appreciated in our culture, is dangerous.

(FGD-1)

#### 4.3.3. Effects on Health

Spending prolonged time on the Internet and online games not only takes away study time but also takes a heavy toll on wealth and well-being. The addiction to games and screens often becomes unstoppable. Playing violent games also affects them emotionally. Parents are highly concerned about the future careers of their children. They think that due to excessive access to the Internet, their children are not attentive to their studies. For example, one parent mentioned:

My kids are less interested in studying and not doing school homework on time, they are not following family routines for daily life activities such as eating and sleeping on time, which will make create problems in their future career, professional and family life. I am very worried, really worried about their future

(Interviewee-8)

Disruption to daily routines, such as eating times and sleeping times, is seen as a hindrance to children growing up and following a disciplined lifestyle. Parents are also concerned about their children’s mental and physical health issues, which they think are the result of excessive internet usage. For example, one typical answer of an interviewee is written below:

They are spending too much time on the Internet and thus getting addicted; I will say addicted to the Internet and other social media, which also affects them emotionally and physically.

(Interviewee-6)

[Their children are] becoming less interested in physical activities that cause health and emotional issues such as eye problems, eating disorder and they are becoming and anti-social.”

(Interviewee-5)

Another response was as below:

I think now our kids are becoming careless about routine—daily life activities such as taking food and going to bed (to sleep) on time. No physical exercises, not playing on the field, that’s bad, very bad!

(Interviewee-10)

### 4.4. Communication, Knowledge and the Generation Gap between Parents and Children

Due to cultural norms and practices, some children are not very friendly to their parents. In response to our question, ‘How often do you talk to your children about their online friends?’, a parent replied, “My daughter who is almost 18, never mentioned if she has any online friends”.

Children of this age are more technologically savvy than their parents, who grew up in a different environment. The research found a significant knowledge gap between parents and children, particularly regarding using the Internet and technological devices. This knowledge gap made parents less confident to manage and control online protection.

The research asked the survey participants if they discuss the risks associated with the use of the Internet with their children. There were three options to answer this question: ‘Yes’, ‘No’, and ‘They know the Internet better than me, so I need not tell them anything’. The result found the highest response rate for ‘Yes’ with 69.90% while the response rate for ‘No’ was 25.8%, and for ‘They know the Internet better than me, so I need not tell them anything’, it was only 4.30% for the overall sample.

Even though some parents have a good background knowledge of IT, they are not very confident about controlling the risks of the Internet. For example, one interviewee said:

Not 100 per cent. Because although I am very much familiar with the IT industry, I have attached myself to the IT industry for about 20 years but for the last one and half years I am not dealing with IT. So, I am pretty much worried now about their involvement in the Internet and other things you know. I’m not 100 per cent sure but 50 or 60 per cent as I have some background in IT. “

(Interviewee-3)

Most of the participants were found to be unaware of the existence of any organisation that gives training to parents about online protection. For example, one interviewee mentioned:

I know about some of the programs such as Norton (an Anti-virus program), but I am not sure about how to use these programs and software to control internet risks.

(Interviewee-8)

Some parents said that parents are also responsible, but the problem is some parents have very limited knowledge of internet use and especially how to control internet risks as they have no technological knowledge.

### 4.5. Ignorance of the Legislation and Policies

The research also asked the sample participants if they were aware of any legislation that safeguards children from the risks of the Internet. About 80% of respondents were found to be not at all aware of any government policies and initiatives regarding children’s online protection. This is of significant concern as the Australian government has invested considerable efforts and multiple initiatives to formulate child safety policies and guidelines through the eSafety Commissioners Office, one of the leading organisations focused solely on this issue.

The research also collected survey data on sample participants who are familiar with the cyber policy of their children’s schools. When this know-how was compared with mainstream Anglo-Saxon respondents, the data showed that this ignorance was most prevalent amongst the migrant and multicultural communities who formed the majority of respondents. Other parent groups were more aware/equipped compared to South Asian ethnic groups. Consistent with the survey result, a lack of awareness about the legislation and policies in relation to children’s online protection was equally evident amongst the FGD and the interview participants. For example:

I think my knowledge is too limited about what’s Government is doing about it. I shouldn’t comment but Government can put some money into research developing different applications that parents can use to put through some potential lock system or some sort of applications. Yeah, Government could do but I don’t know whether Government has done that yet or no because I haven’t explored those options yet.”

(FGD-2)

Parents were found to be ignorant about school policies as well. When asked specifically about school policies, many answered: “No, I don’t know. This is my weakness”; “No, I can’t remember,” and so on. However, some parents have shown firm trust in the school, according to one:

Yes, I am happy with the school policies. I know that according to the school policies students are allowed to visit the websites that are good and not the other sites in school. That is good.

(Interviewee-4)

More than 60% of parents who responded are unable to assess or are unsure how to assess the danger posed by internet use.

I have no idea and no capacity to control them … But I heard from them (children) that they learnt from school how to deal with the risks. I think they know at least something to control and deal with the risks.

(Interviewee-5)

Because the Internet is not new to me and I use the Internet, but you can’t say what your children are doing in their bedrooms? What they are doing under their quilt? I thought that honey (son) is sleeping and when I just remove his quilt, he was watching a movie. Just a normal movie, not a bad movie. But when I was young there were no tablets, no mobiles. So, we had to watch a movie on TV nowadays they are not interested to watch a movie on TV. They can use their devices in their beds.

(Interviewee-12)

### 4.6. Strategies to Prevent the Risks

The survey results found that most parents believe that explaining the danger of the Internet to their children is the most popular way to mitigate the risks of the Internet, as found with 62.25% of responses for the overall sample participants. The qualitative research also gathered participants’ views and best practices to mitigate online risks for children, which have been summarised into three categories discussed below:

#### 4.6.1. Role of Government

Many parents want to see their government be more proactive in the area, both in policy formulation and in creating more awareness and training for parents. Some parents consider this a nationally important issue, and the government should provide training on parental control at home with the help of local schools and community organisations. Some parents, mainly from developing countries, have very limited knowledge of technical issues around using and controlling the Internet. Some parents think the government is not doing enough to tackle this problem as they are with other major global issues, such as climate change, smoking, etc. As one parent said:

I would say the government is putting probably a lot of Ads for people who are smoking to quit smoking. They are putting a lot of effort into it, a lot of advertising. I think they could Ad these kinds of things to advertisements making people aware saying that “Hang on, your kids are using the Internet, and this is a risky beast to deal with so you gotta be careful how they use it. If you need help as you know Government says you need help, dial this quit number on how to quit smoking. So, what on they put dial number on there where Government can put together professionals who can provide advice these are the things available. The person who does never use the Internet in their life how he is gonna control his kid’s use Internet? So obviously he needs a helpline where Government can provide help. When they call the helpline Government can provide that they have a key area using that there are applications available, do you use that and this is how they can use them? I guess providing a bit of awareness, and advertisement could help

(Interviewee-13)

The government’s role in filtering and preventing access to inappropriate materials such as pornography and violent games has also been emphasised. Many parents were found to be in favour of the filtering and blocking sites by the government that could affect children mentally and physically. According to one parent:

Look I do not know how feasible it is, but I think Government can make a bit strict on the use of the Internet. For example, rather than filtering from our side—I know about a few countries I am not telling the name, but there are a lot of bars involved … I understand you are restricting freedom but considering this particular case of child security while using the Internet I would suggest that if the government can take this kind of action, it might be very helpful

(Interviewee-5)

Parents emphasise making businesses more accountable for their design and delivery of IT services online to protect children from the risks and harmful effects of internet use.

#### 4.6.2. Role of Parents

Parents are convinced that their role is critical and feel they should give more attention and care to their children’s online use. Parents’ mediation role has been discussed widely in recent studies (Jeffery, 2021). According to one parent:

First and foremost, many of the things come from your family … I would say work together with the children to develop awareness in the mind of the children and I believe that you should be friends—you should maintain friendship relation with your children. So that whatever they want to share they have the freedom to share with you and that is how you know best what’s in their minds and what they are involved in.

(FGD-2)

Experience suggests giving more time and taking children out to the playground will have a far more significant outcome in regulating children’s online behaviours. A disciplined and agreed conducive culture that was participated in by all adults and children on the Internet at home was found to be effective in minimising risks and overuse. As a respondent said:

So, the same rule applies to the parents. Ignorance is not the law of excuse. If your kids are using the Internet, you should train yourself or if you don’t know, you are not technically savvy, you go and ask a professional like when you got sick you go and ask the doctors. There are professionals out there who develops these sort of thing, protection mechanism, go ask them, and get help

(FGD-3)

The result has been echoed in interview data too. Such as:

They discuss every time, and we have our dinner or lunch together … This is our gathering time when we sit together, and discuss the present and the future. For them what they are doing … what they are doing, and what kind of videos they are watching.

(Interviewee-3)

They discuss that they are watching some political and religious and sporting and different kinds of videos and they discuss with me and with my wife that they are watching this kind of thing.

(Interviewee-4)

I guess Government is responsible to make it accessible for those parents who are not technically savvy. That’s the best Government can do but remember that ignorance is not the law of excuse. It’s not excused—there is no excuse for not seeking help. If you get sick, you go to the doctor. What services need to develop I think that’s where Government come into play.

(Interviewee-12)

I keep them involved in some kinds of outdoor activities. Just like today, we wash our car and our garage. They said why don’t you take your car to the service station? I say no, it’s our car and we can wash this car and it creates a love between us and our car. So, I involve my children in different kinds of external activities.

(Interviewee-3)

Some parents emphasise some easy technical solutions that should be available in the market for easy implementation. One parent said that businesses should be able to guide parents on technical matters on parental control, with filters when they buy internet solutions, routers, etc.

Some parents were found to be overly concerned and in favour of more restrictive mediation by enabling parental locks in applications available for different age groups or giving them a separate internet zone. According to one parent:

I think we should not allow them to use computers and laptops in their bedrooms. They should use a computer an open space and then we can watch and monitor what they are doing. In addition, we can check their internet browsing history. We can also be a friend on their Facebook account and can check what they are doing and who their friends are, and we can track their Facebook activities.

(FGD-1)

Parents do not have any time to go with the training because it is very simple and easy nowadays for every parent. If I go and just get a new washing machine, an automatic washing machine and I do not have any time to read a manual. I asked the salesperson so, what are the features? How can I use that? Therefore, the people who are selling either these kinds of internet facilities tablets surface or any kind of devices, which is used with the Internet, just give some filters or parental guidance at the time of selling. These two or three are prime just read it and then use them according to these filters or devices in your home.

(Interviewee-7)

## 5. Discussion Overall

The results of this research demonstrate that parents’ perception of the online risks their children face is heavily influenced by their personal experience and understanding of the nature of online threats and the Internet in general. Demographic factors, including the ethnicity of participants, also played a role in influencing this perception of risk.

Two themes relating to parents’ perception of risk have emerged from the data collected. Firstly, parents are not well aware of the range and scope of cyber threats and, therefore, often make false assumptions about the risks to their children. Secondly, personal bias, ethnicity, and cultural settings can influence parents’ perception of the Internet and its risks. These trends indicate an overall parental ignorance of the nature of cyber threats and how these threats can be mitigated for the protection of their children in certain ethnic communities.

Risks such as online gambling, buying and selling of stolen goods, cyber-attacks, and identity theft were not identified by any research participants as threats. This demonstrates a significant lack of parental awareness of the range of cyber risks that are relevant to their children and a narrow focus on specific cyber threats, such as watching inappropriate content and loss of security.

Furthermore, approximately half of the parents were unaware of effective responses to cyber threats and/or did not talk to their children about internet safety, demonstrating that a significant portion of parents do not have the skills and awareness to effectively address and mitigate cyber risk. Less than half of the parents in the survey believed that they could assess the danger posed by the Internet. This research has demonstrated that while parents are concerned with some areas of cyber risk concerning their children, they lack the breadth of knowledge to identify and mitigate all cyber threats. This research also demonstrates that parents do not have a detailed understanding of the range and extent of cyber threats; instead, they are more likely to identify more visible threats, including watching inappropriate content online, losing privacy, and cyberbullying.

There is a simultaneous battle between the development of security to protect children from risk and the evolution of online threats. Online abusers will keep exploiting weaknesses, whether social or technical. In addition to the technological tools families can employ, the best defence is critical thinking—understanding when things are too good to be true or knowing to pause for a few seconds to consider the consequences of personal actions online. Parental awareness is being disrupted by the increased privatization of internet use (e.g., smartphones) and thus may contribute to this lack of parental knowledge.

Technology is an integral part of modern society and cannot be effectively avoided. Every possible risk associated with technology cannot be eradicated but by taking appropriate strategies and reasonable precautions, the risks can be greatly reduced. Subsequently, parents must have an adequate understanding of the risks affecting their children online and know how to recover if something does go wrong. This will help the parent to pass the baton of civilisation to the next generation smoothly, as has occurred in the past. The cost of not doing anything in this area could be very high for society and the future we are yet to envisage. The results of this study suggest the need for appropriate mediation strategies to reduce the risks facing children online, with a specific focus on the context and culture of these solutions. The mitigation strategies will need to be designed to maximise their successful adoption by all demographic and ethnic groups.

The most important task, to start with, should be to make parents aware and equipped to raise their children in this digital age through appropriate interventions, training, and programs. One objective is to bridge the generational and communication gaps so parents remain ahead of the game and can discharge parental duties without ignorance, fear, and uncertainty of the digital era. The socio-economic impact of the ignorance of cyber and safety skills of parents, teachers, and caregivers needs to be assessed first, based on which interventions can be planned and developed to fill the gap. Such programs should be designed not only to bridge a better understanding and promote positive relationships but also to foster self-esteem among children. The generational shift between digital natives and millennials needs to be managed and mitigated through strategies such as open communication between children and parents regarding their online activities.

Protective factors include positive attention from parents, supportive relationships with other adults, and extended family, family harmony, and religious faith. These need to be considered, especially in how they may play a role in certain ethnic and cultural communities, including Aboriginal and Torres Strait Islanders. There is a conflict between the protective factors associated with traditional practices and the risk factors arising from poverty and destitution in specific demographic and cultural communities, including Aboriginal and Torres Strait Islanders.

## 6. Conclusions

This study has painted a rich picture of the perception of multicultural parents and presented the diverse views expressed by parents. The mixed methods study compared the survey results with qualitative findings and complemented the statistical findings through more in-depth explanations and analyses of the qualitative data. The study established the role of ethnicity and culture in parental perceptions of online behaviour issues, where there was a clear gap in research [14]. Similar studies conducted on this issue [2,19,20,26] did not consider or highlight ethnicity as a factor in online usage by children or how their parents perceive their children’s usage, including potential threats. This study fills the gap in scholarly debate. Existing and recent tools and practices need to be reviewed and re-examined with a fresh outlook through multiple lenses to accommodate the changing landscapes of society and technology. For example, privacy statements and legislation around online interactions often fail to capture the status quo and do not necessarily encourage safe cyber activity.

Considerable work is needed to incorporate safety measures within children’s toys, particularly online games. Online games significantly influence children’s behaviour and aptitude while growing up in the digital era. The potential for developing educational toys and devices to mitigate the cyber risks facing children also needs to be expanded and encouraged by the relevant authorities and schools.

This study will help researchers and policymakers to formulate appropriate policies and strategies based on prevailing perceptions among parents, particularly from multicultural communities.

### Limitations and Future Research

This research is a snapshot of parents of specific ethnicities and does not capture the broad spectrum of communities represented by a large population. Potential biases may also arise from the focus groups and interview participants, as they involve specific ethnicities and communities and so may not reflect an accurate sample of the population.

However, the snapshot of these 204 parents from three major cities (Sydney, Melbourne, and Canberra) of Australia, followed by a qualitative enquiry, provides valuable insights into the phenomenon and a basis for designing broader research, utilising its findings as the starting point.

Future research should investigate the role of ethnicity and culture in parental perception, risk assessment and categories, and effective remedial strategies and interventions to bridge parents’ awareness and knowledge gaps. Future studies should also look at the evolving technologies and their effects on children and their relationships with parents and society, both in the short and long term. Future studies may dig deeper into the issue to see the moderating effect of various components of ethnicity and culture.

## Figures and Tables

**Table 1:** Survey questionnaire overview.

Section	Indicators	Purpose
Awareness of the Internet	Definition of the Internet, daily internet use, importance of access, importance of updating knowledge	Description of parents’ internet use, attitudes and skills
Risk Perception	Belief in risk, classification of risk, experience of risk, factors influencing risk, ability to assess danger, understanding of cyberbullying, ability to respond to threats	Description of parents’ perception of risks associated with internet use
Parents’ internet beliefs	Time children spend online, permission agreements, children’s skill levels, knowledge of internet use, monitoring practices, mitigation techniques, definition of excessive use, cyber policy and legislation awareness, willingness to undergo training, confidence level	Description of parents’ beliefs about their children’s internet use and the role they play in mitigating risk
Demographics	Age, gender, children’s age and gender, ethnicity, place of birth, number of years in Australia, highest level of education completed, medium of educational instruction, region in Australia	Description of samples

**Table 2:** Background characteristics of the participants and their percentage distribution by the Composite Risk Perception Index from selected ethnic/study groups/areas in Australia (n = 200).

Characteristic and Categories	Observations No. (%)	Composite Risk Perception Index (CRPI)			
		No Risk	Some Risk	Real Risk	*p*-Value
Socio-demographic					
Ethnicity					0.023
South-East Asian	97 (48.5)	46.2	32.7	54.8	
Others	103 (51.5)	53.8	67.3	45.2	
Sex					0.001
Male	104 (52.0)	76.9	69.2	43.0	
Female	96 (48.0)	23.1	30.8	57.0	
Highest level of education					0.054
Not finished high school	33 (16.5)	30.8	26.9	11.1	
High school to college diploma	34 (17.0)	7.7	15.4	18.5	
Undergraduate to Doctoral degree	133 (66.5)	61.5	57.7	70.4	
Parents access the Internet using iPad					0.012
No	120 (60.0)	61.5	76.9	53.3	
Yes	80 (40.0)	38.5	23.1	46.7	
Knowledge about cyber risks					
Parents understand cyberbullying					0.001
No	37 (18.5)	53.8	23.1	13.3	
Yes	163 (81.5)	46.2	76.9	86.7	
Familiar with School’s cyber policy and Australian legislation					0.028
No	85 (42.5)	7.7	48.1	43.7	
Yes	115 (57.5)	92.3	51.9	56.3	
View of appropriate internet use by children					
Purpose of use (Contact friends or families)					0.028
No?	49 (24.5)	46.2	32.7	19.3	
Yes?	151 (75.5)	53.8	67.3	80.7	
Reasons children should access the Internet (Game)					0.038
No	139 (69.5)	38.5	69.2	72.6	
Yes	61 (30.5)	61.5	30.8	27.4	
Parental view of dangers on the Internet					
Believe Internet exposure to danger/risk					0.000
No	12 (6.0)	38.5	7.7	2.2	
Yes	188 (94.0)	61.5	92.3	97.8	

**Table 3:** Regression coefficients and odds ratios for the likelihood of moderate and severe forms of Composite Risk Perception of cyber risks faced by children by some selected significant characteristics.

Characteristic	Composite Risk Perception Index (CRPI) ^a^					
	Some Risk			Real Risk		
*Socio-Demographic*	B	Odds Ratio	95% CI	B	Odds Ratio	95% CI
Ethnicity	−0.222	0.80	0.12–5.12	1.787 *	5.97	0.95–37.24
South-East Asian						
Others ^b^						
Sex	−0.459	0.63	0.09–4.43	−1.916 **	0.15	0.02–0.87
Male						
Female ^b^						
Highest level of education	1.807 * 1.391	6.09 4.02	0.73–51.13 0.20–82.99	1.071 1.595	2.92 4.93	0.35–24.26 0.23–95.17
Not finished high school						
High school to college diploma						
Undergraduate to PhD ^b^						
Parents access the Internet using iPad	1.963 **	7.12	1.21–42.04	1.037	2.82	0.51–15.57
No						
Yes ^b^						
*Knowledge about cyber risks*						
Parents understand cyberbullying	−2.196 **	0.11	0.02–0.79	−2.541 ***	0.08	0.01–0.53
No						
Yes ^b^						
Familiar with School’s cyber policy and Australian legislation	2.459 **	11.698	1.07–128.03	1.886	6.59	0.63–69.27
No						
Yes ^b^						
*View of appropriate internet use by children*						
Purpose of use (Contact friends or families)	−0.954	0.39	0.07–2.06	−1.472 *	0.23	0.04–1.21
No						
Yes ^b^						
Reasons children should access the Internet (Game)	1.417 *	4.12	0.80–21.20	1.531 *	4.62	0.94–22.69
No						
Yes ^b^						
*Parental view of dangers on the Internet*						
Believe Internet exposure to danger/risk	−1.793 *	0.17	0.02–1.33	−2.761 ***	0.06	0.01–0.53
No						
Yes ^b^						
Model fitting information:						
−2 Log Likelihood			174.50			
Chi-squared (df)			82.23 (20) *****			

^a^ Reference category of the dependent variable is no risk of children. ^b^ Omitted categories (i.e., reference class for each independent variable) not shown. * *p* < 0.10; ** *p* < 0.05; *** *p* < 0.01; ***** *p* < 0.0000.

## Data Availability

The data presented in this study are available on request from the corresponding author. The data are not publicly available due to ethical restrictions.

## Appendix A

**Semi-Structured Question Protocol and Guide**

**MAIN QUESTIONS**

The first set of questions will try to understand the parental perception of risk associated with ubiquitous access to the Internet; the second set of questions is associated with the strategies adopted by parents to protect their children from risks accompanying internet use. To protect children from the risks encountered on the web, the first sub-set of questions will try to uncover parents’ awareness of their child’s internet behaviour. The second sub-set of questions will try to gather parental knowledge of actions to be taken to manage the threat, and the last sub-set of questions will try to understand their ability to implement actions to alleviate those threats. Because of the nature and scope of the study, it is expected that relevant supplementary questions may arise based on the response of the participant.

**RELATED TO PARENTAL PERCEPTION OF RISK ASSOCIATED WITH INTERNET USAGE**

A. **Perception of risk**
(1) What do you understand by the Internet?
(2) Is access to the Internet necessary?
(3) Do you think there is a risk in accessing the Internet?
(4) What is the risk?
(5) How do you know about this risk?
(6) Do you think the risk is less or more for children? Why?
(7) Do you think the risk is dependent on the age and gender of the children? Why?
(8) How much use of the Internet is too much? Why did you set this limit?

**RELATED TO PARENTAL STRATEGIES TO PROTECT CHILDREN FROM THE RISKS ARISING OUT OF INTERNET USE**

B. **Parental Awareness of their Child’s Internet Use**
(1) How much are you aware of your children’s online behaviour? Do you know what your children do on the Internet? How?
(2) Do your children talk about their online activities?
(3) To what extent do you expect your children to tell you about their online activities?
(4) Are you aware if your children have made friends online?
(5) How often do you talk with your children about their online friends?
(6) How often do you talk to your child about what you do online?

C. **Parental Knowledge of Actions that Lower Risks Associated with Internet Use**
(1) What do you think are some of the risks associated with use of the Internet?
(2) Do you think adults and children are equally at risk? Why or why not?
(3) Do you worry about your children being exposed to these risks?
(4) Do you think your children are capable of identifying these risks?
(5) How did you come to assess your children’s capability or skill on the Internet?

D. **Parental Ability to Implement Actions to Eliminate Risks Associated with Internet Use**
(1) Do you think you can reduce or remove some of the risks arising out of internet usage?
(2) How do you address some of these threats?
(3) Are you familiar with any technology that can help with eliminating these risks?
(4) Are you familiar with your child’s school policies regarding keeping safe on the Internet?
(5) Are you aware of any legislation that can help you effectively reduce some of the risks? What do you think are some of the risks associated with use of the Internet?

**CONCLUSIONS**

(1) Do you have any questions or any other related comments before we end?
